# Age Dependent Changes in Corneal Epithelial Cell Signaling

**DOI:** 10.3389/fcell.2022.886721

**Published:** 2022-05-05

**Authors:** Kristen L. Segars, Nicholas A. Azzari, Stephanie Gomez, Cody Machen, Celeste B. Rich, Vickery Trinkaus-Randall

**Affiliations:** ^1^ Department of Pharmacology, School of Medicine, Boston University, Boston, MA, United States; ^2^ Department of Biochemistry, School of Medicine, Boston University, Boston, MA, United States; ^3^ Department of Ophthalmology, School of Medicine, School of Medicine, Boston, MA, United States

**Keywords:** cornea, stiffness, live cell imaging, cell-cell communication, calcium mobilization

## Abstract

The cornea is exposed daily to a number of mechanical stresses including shear stress from tear film and blinking. Over time, these stressors can lead to changes in the extracellular matrix that alter corneal stiffness, cell-substrate structures, and the integrity of cell-cell junctions. We hypothesized that changes in tissue stiffness of the cornea with age may alter calcium signaling between cells after injury, and the downstream effects of this signaling on cellular motility and wound healing. Nanoindentation studies revealed that there were significant differences in the stiffness of the corneal epithelium and stroma between corneas of 9- and 27-week mice. These changes corresponded to differences in the timeline of wound healing and in cell signaling. Corneas from 9-week mice were fully healed within 24 h. However, the wounds on corneas from 27-week mice remained incompletely healed. Furthermore, in the 27-week cohort there was no detectable calcium signaling at the wound in either apical or basal corneal epithelial cells. This is in contrast to the young cohort, where there was elevated basal cell activity relative to background levels. Cell culture experiments were performed to assess the roles of P2Y2, P2X7, and pannexin-1 in cellular motility during wound healing. Inhibition of P2Y2, P2X7, or pannexin-1 all significantly reduce wound closure. However, the inhibitors all have different effects on the trajectories of individual migrating cells. Together, these findings suggest that there are several significant differences in the stiffness and signaling that underlie the decreased wound healing efficacy of the cornea in older mice.

## Introduction

The physical changes undergone by tissues and cells with age or disease can reflect changes in the mechanical forces present in the tissue, leading to functional aberrations in processes including wound healing. Such changes are detected in endothelial vasculature (Derricks et al., 2015), bone remodeling (Wang et al., 2018), and cell-cell communication between vascular endothelial cells (Kohn et al., 2015). The cornea is an excellent tissue model to study such forces, as it is exposed to the environment and subject to mechanical forces such as shear stress from blinking. This force may change with the quality of tear film, resulting in alterations in cell-cell junctions. Additional mechanical forces can be generated by changes in extracellular matrix from wound healing. Although the method in which the corneal epithelium modulates force generation is unknown, the corneal epithelium adheres to its basement membrane through hemidesmosomes, a complex of proteins that modulate force generation in skin (Wang et al., 2020).

The integrity of the multi-layered epithelial sheet depends on a complex of cell-cell junctions including tight junctions, desmosomes, actin-based cell junctions and gap junctions that are present in unwounded and wounded tissue. Several studies have shown that mechanical characteristics of the substrate can ultimately influence the phenotype of the overlying cells (Engler et al., 2006). When epithelial cells are cultured on substrates of different stiffnesses, distinctly different sized focal adhesions were detected (Onochie et al., 2019). These may correlate with earlier studies in rat corneas demonstrating an overall increase in vinculin (22-fold, 18 h post-injury), where this focal adhesion protein was localized to basal cells at the leading edge of the wound (Zieske et al., 1989). In addition, an increase in surface stiffness reduces the number of vinculin focal adhesions along the leading edge of the migrating epithelial cells. These alterations in expression, in addition to changes in phosphorylation of vinculin, suggest that its presence plays an important role in the cellular migration process by interfacing between the cytoskeleton and the extracellular matrix (Onochie et al., 2019).

There are numerous studies examining how single cells or clusters of cells change shape and exert force (Gardel et al., 2010; Tambe et al., 2011; Brugues et al., 2014; Mak et al., 2016). These forces are thought to occur through the presence of lamellipodial protrusions at the leading edge of the wound, which are associated with the assembly and disassembly of focal adhesions (Peyton et al., 2007; Petrie et al., 2012). However, there is minimal data explaining how multi-layered epithelial sheets exert force and migrate after an injury, and how these forces change with age. One of the first studies examining traction forces exerted by sheets demonstrated that it generates unique forces on the underlying basement membrane that depend on the stiffness of the substrate, with significantly greater retraction on a substrate of 8 kPa compared to stiffer substrates (Onochie et al., 2019). Investigations on single cells support these studies and demonstrate that the composition and the rigidity of the basement membrane play a critical role in the signaling that underlies cell adhesion, migration, and differentiation (Discher et al., 2005; Mammoto and Ingber, 2010; Sazonova et al., 2011).

The composition of the corneal epithelial basement membrane is known to change with injury, age and/or during certain pathologies such as diabetes. (Torricelli et al., 2013). For example, fibronectin is not detected in the unwounded basement membrane but after injury it is present transiently along the basement membrane and throughout the stroma (Blanco-Mezquita et al., 2011; Lee et al., 2014). The increase in fibronectin is attenuated when the cornea is exposed to hypoxia (Onochie et al., 2019). Matrix proteins can also be secreted by the stroma in response to inflammation and alter the rigidity of the substrate underlying the epithelium (Nowell et al., 2016). Thus, the change in the availability or presence of a matrix protein may alter wound healing ability.

Furthermore, the localization and regulation of two ion channel proteins, P2X7 and pannexin-1, have been of great interest in respect to their response to injury. Both proteins exhibit injury induced changes in localization, and the intense staining that is adjacent to the injury corresponds to the region of intense calcium mobilization or signaling between cells (Lee et al., 2019; Rhodes et al., 2021). Inhibition of either P2X7 or pannexin-1 greatly attenuates calcium signaling events, cellular motility, and migration of the epithelial sheet (Lee et al., 2019). In addition, inhibition of P2X7 has been found to inhibit turnover of actin within the cell (Minns et al., 2016). As these channels have been shown to play a role in both signal transduction and in cytoskeletal rearrangement, we are intrigued by their role in mediating cytoskeletal-ECM interactions.

We hypothesize that changes in tissue stiffness of the cornea with age may alter the calcium signaling between cells after injury, and the downstream effects of this signaling on cellular motility and wound healing. Nanoindentation of the cornea was used to examine changes in stiffness with age. Live cell imaging on young and aged mouse corneas was used to monitor calcium signaling in central and limbal regions of the epithelium after injury, and to quantify wound healing over time in both live corneas and in cell culture. Signaling events throughout the cornea were identified using MATLAB Kymograph and Detected Events algorithms.

## Methods

### Animals

C57Bl6 mice were purchased from Jackson Labs between the ages of 9 and 27 weeks. Young mice are defined as being between 9 and 13 weeks of age. Old mice are defined as being 27 weeks old. Mice were euthanized and a minimum of three eyes from different mice per condition were analyzed. The research protocol conformed to the standards of the Association for Research in Vision and Ophthalmology for the Use of Animals in Ophthalmic Care and Vision Research and the Boston University IACUC protocol.

### Cell Culture

Human corneal limbal epithelial (HCLE) cells, a gift from Dr. Gipson (Schepens Eye Research Institute/Mass. Eye and Ear, Boston, MA, United States) were verified at Johns Hopkins DNA Services (Baltimore, MD) and used as described (Lee et al., 2019). Cells were maintained in Keratinocyte Serum-Free Medium (KSFM) with the following growth supplements (25-μg/ml bovine pituitary extract, 0.02 nM EGF, and 0.3 mM CaCl_2_). For live cell imaging assays, the cells were plated on glass bottom dishes (MatTek Corporation, Ashland, MA, United States) and 16–24 h before experimentation, the medium was changed to unsupplemented KSFM as described (Lee et al., 2019).

### Cellular Migration

For cellular mobilization experiments on cultured cells, HCLE cells were plated on a p35 MatTek well (MatTek Corporation, Ashland, MA, United States) and grown to confluence. For inhibitor experiments, confluent cells were pre-incubated with either ArC 118925XX (Tocris, Minneapolis, MN, United States), A438079 (Tocris), or 10PanX (Tocris) for 1 h to inhibit P2Y2, P2X7, or pannexin-1 respectively. Cells were pre-incubated with SiR-Actin (Cytoskeleton, Inc., Denver CO, United States) (1:1,000) counterstain for 20 min at 37**°**C. Excess stain was rinsed. Imaging was performed using a Zeiss Axiovert LSM 880 confocal microscope (Zeiss, Thornwood, NY, United States) utilizing the ×20 objective and the environmental chamber maintained an environment of 37°C and 5% CO_2_. Images were collected every 5 min for 4 h. FIJI/ImageJ (NIH, Bethesda, MD; http://imagej.nih.gov/ij/) region of interest analysis was used to quantify wound perimeter over time and calculate the percent wound closure at each time point. FIJI/ImageJ centroid analysis was used to plot the movement of individual cells both parallel to and perpendicular to the wound to calculate their trajectories.

### 3D Printing of Corneal Holder

**FIGURE 1:**
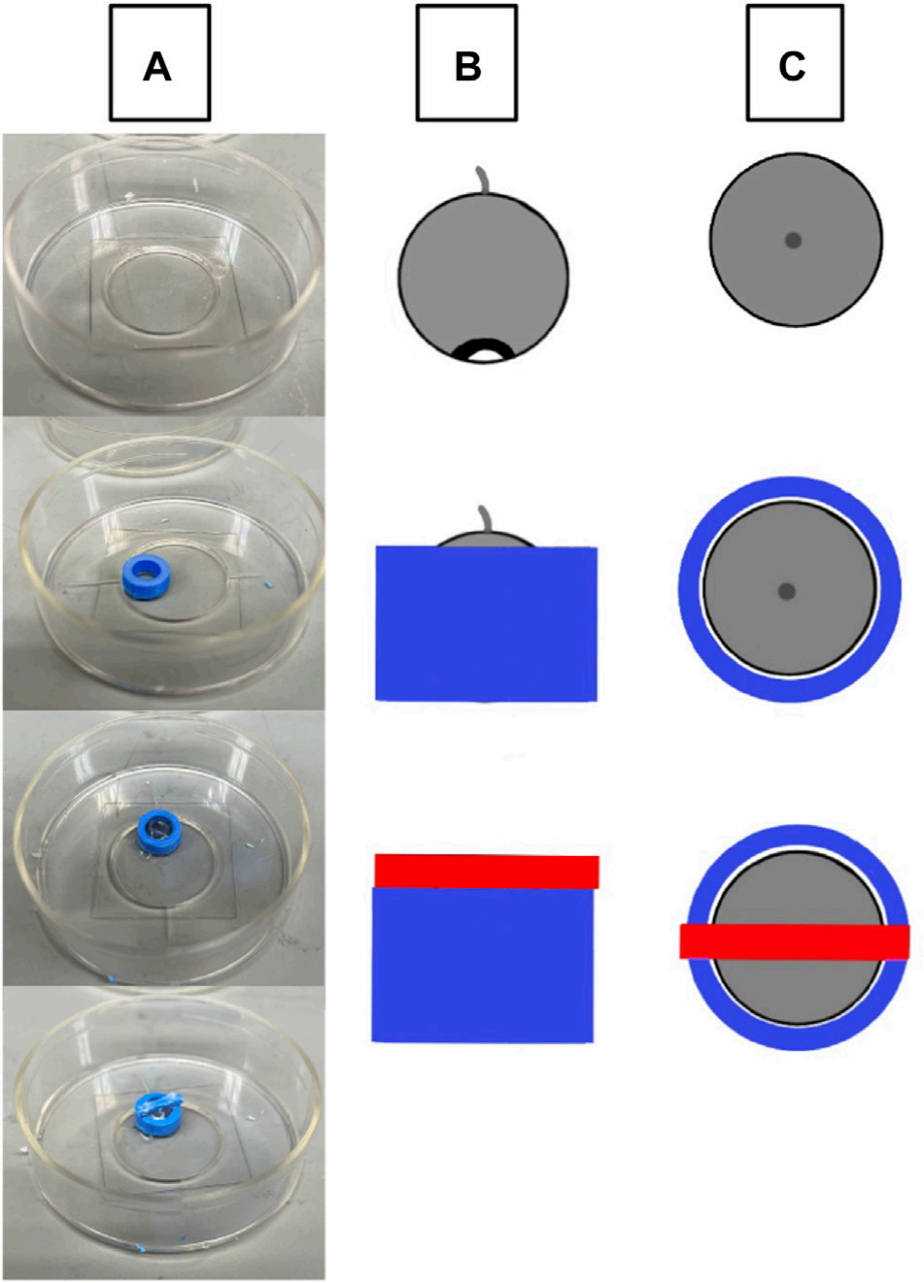
Schematic of eye stabilization using a 3D printed holder. **(A)** A 3D printed holder (blue) is adhered to a MatTek p35 cover slip, the intact globe is placed within the holder with the cornea facing down, and the cover is placed over the center of the eye and adhered to the holder. **(B)** A lateral view of eye orientation (grey circular object) and the relative sizes of the holder (blue) and weighted cover (red). **(C)** The top-down view of the holder setup.

3D printed holders (Figure 1) were created using an Ender 3 Pro 3D printer (Shenzhen, China) using 1.75 blue polylactic acid (PLA) plastic. Holders were made assuming that the diameter for a mouse cornea ranges from 2.3–3.5 mm depending on the age and mouse strain (Henriksson et al., 2009). The final size of the holder where the globe was stabilized had an inner diameter of 3.6 mm at the widest point and was created with the software Autodesk Fusion 360 (San Rafael, CA, United States). The holder was adhered to a cover slip, the globe was placed in the holder as appropriate for imaging or nanoindentation and bathed in medium to prevent drying.

### Nanoindentation

Corneal epithelium and stromal stiffness were measured using a PIUMA Nanoindenter System from Optics 11 (Amsterdam, Netherlands) with probes from Optics 11 (Cambridge, MA, United States). Measurements were performed by placing the intact globe into the 3D printed holder, cornea side up, and bathing the eye in serum-free keratinocyte medium to avoid dryness. The probe was lowered onto the center of the cornea and measurements were taken both at the center of the cornea and at 5 micron increments in the *x* and *y* directions. The stromal stiffness and basement membrane were measured by abrading the epithelium. Previous published studies using electron microscopy have shown that this protocol produces a clean abrasion (Payne et al., 2000).

Optimization experiments for corneal measurements revealed that the most reliable results came from using a stiffer probe for the epithelium and a softer one for the basement membrane; epithelium: stiffness of 4.41 N/m with a tip radius of 20.5 mm and basement membrane: stiffness of 0.24 N/m with a tip radius of 28.5 mm, as suggested in contact with the manufacturer. Measurements for the epithelium and basement membrane were taken using the Oliver and Pharr Model. As the probe may adhere slightly to the surface, use of this model is critical. The Effective Young’s Modulus was calculated by the PIUMA software when the Poisson ratio was unknown.

### Live Cell Imaging

Calcium mobilization experiments were performed as described previously (Klepeis et al., 2001; Weinger et al., 2005; Lee et al., 2019). The imaging of intact murine eyes was modified from that of corneas as described (Lee et al., 2019; Rhodes et al., 2021). Globes were enucleated and were preincubated in Fluo-4, AM (1:100) and CellMask™ Deep Red (Thermo Fisher, Waltham, MA, United States) (1:10,000) with a final concentration of 1% (v/v) DMSO and 0.1% (w/v) pluronic acid for 2 h at 20°C. Excess dye was washed. Puncture wounds were performed with a 25 gauge needle. To maintain consistency the same person made the injuries. Wounds were on average 10.67 μm ± 1.17 μm deep and 30.53 μm ± 13.01 μm diameter. The wound depth and diameter did not vary significantly between samples (two-tailed Student’s t-test; p = 0.999 for both depth and diameter). A 3D printed holder was adhered to the glass bottom p35 Mat-Tek dish using sterile glue. The globe was placed within the contours of the 3D printed holder with either the central cornea facing down or corneal-limbal interface facing down depending on the parameters of the experiment. A weighted 3D printed cover was placed over the eye and holder to stabilize the globe. Images were collected every 3 s for 45 min on a Zeiss Axiovert LSM 880 confocal microscope (Zeiss, Thornwood, NY, United States) utilizing the ×20 objective for signaling experiments and every 5 min for 4 h for longer-term migration experiments. Z-stacks were taken through the corneal epithelium. MATLAB (MATLAB, MathWorks, Inc.) scripts were used to identify and quantify signaling events, and FIJI/ImageJ (NIH, Bethesda, MD, United States) was used to measure wound circumference.

### Modeling of Calcium Mobilization

Calcium mobilization is analyzed as F/Fo, which yields an averaged value of the signaling events in a frame over time (Trinkaus-Randall et al., 2000; Klepeis et al., 2001; Weinger et al., 2005). Fo is the baseline signaling intensity before any stimulus has been added to the system and F is the intensity of an image at a given time point. If the F/Fo value exceeds a pre-set threshold determined through prior experimentation, (Lee et al., 2014), a calcium mobilization event is generated. This form of analysis has the disadvantage of looking at an entire 512 × 512 pixel image, and does not permit analysis of the changes in intensity of individual cells within an image over time and space. Identifying the signaling profiles of each cell over time is essential for assessing cell-cell communication *via* calcium signaling. To analyze the spatiotemporal communication between cells after injury, custom MATLAB (MATLAB, MathWorks, Inc.) were developed to identify individual cells within a video. The script quantified intensity of each individual cell over time. Patterns of calcium mobilization were analyzed for individual cells, between clusters of cells, or for the entire population of cells within the video (Derricks et al., 2015). This MATLAB script was modified by Lee et al. (2019) to evaluate clusters of calcium signaling events between cells in specific regions, and to predict the probability of communication between neighboring cells. Kymographs of raw data were generated to represent signaling of individual cells over time, and MATLAB detected events showed the exact time at which signaling events for individual cells in the population occur. In this study, we optimized the script to analyze spatial and temporal signaling events occurring between cells in different Z-planes. This allows us to quantify signaling between basal cells and apical cells, and between basal cells and underlying nerves.

### Immunofluorescence and Confocal Microscopy

Globes were fixed in freshly prepared 4% paraformaldehyde in PBS for 20 min at room temperature. Immunofluorescent staining was performed (Minns et al., 2016; Rhodes et al., 2021). The cornea was removed and cut into radial sections. Briefly, tissue was permeabilized with 0.1% v/v Triton X-100 in PBS for 2–5 min and blocked with 4% BSA in PBS for 1 hour. Corneas were incubated in a primary antibody solution overnight at 4°C. Anti-pannexin-1 polyclonal rabbit antibody directed against pannexin-1 (Cat. #ACC-234) was purchased from Alomone Labs (Jerusalem, Israel). An Alexa Fluor-conjugated secondary antibodies (Invitrogen, Carlsbad, CA, United States) was used at a dilution of 1:300 in blocking solution for 1 h at room temperature. The primary antibody was excluded from the secondary control tissue. The tissue was mounted using VectaSHIELD (Vector Labs, Burlingame, CA, United States). Images were taken on a Zeiss LSM 880 confocal microscope. Fluorescent gain levels were set using secondary control samples and were not changed, and the pinhole size was kept at 1 Airy Unit across all images.

### Statistical Analysis

Values were obtained by taking the mean and standard error of the mean from at least three independent experiments. Depending on experimental parameters, statistical significance was determined with either unpaired or paired two-tailed Student’s t-tests using Microsoft Excel (Microsoft, New York, NY, United States) to compare experimental groups to the appropriate control.

## Results

### Stiffness of the Corneal Epithelium Increases With Age in C57Bl/6J Mice

**FIGURE 2:**
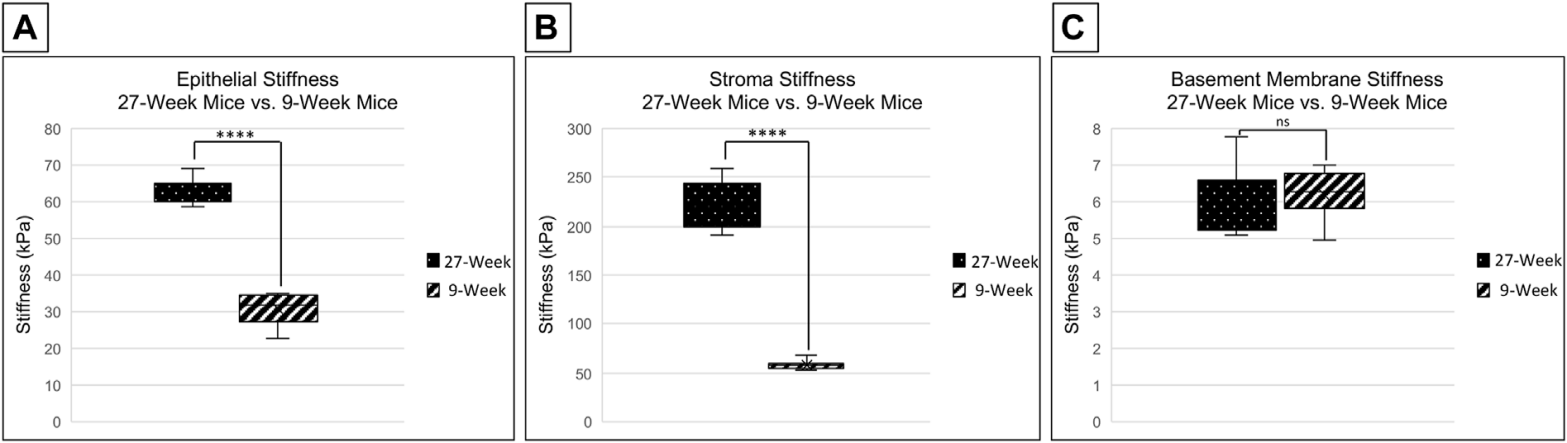
Comparison of corneal stiffness between 9- and 27-week mice. Nanoindentation was performed to compare the stiffness of the corneal epithelium, stroma, and basement membrane between enucleated globes from 9- and 27-week mice. Significance was determined by a two-tailed Student’s t-test comparing epithelium [**(A)** ****p < 0.0001], stroma [**(B)** ****p < 0.0001] and basement membrane [**(C)** not significant, (*p* = 0.506)] between 9- and 27- week mice. The data represent four eyes per condition, with each eye from one mouse.

Changes in stiffness with age were examined in C57Bl6 mice in several regions of the cornea. This was achieved through the use of a PIUMA Nanoindenter System from Optics 11 (Amsterdam, Netherlands). Measurements were performed after placing the eye into the 3D printed holder, cornea side up, and bathing the eye in serum free keratinocyte medium to avoid dryness. The stiffness of the corneal epithelium in 27-week mice is significantly higher than that of 9-week mice, with mean stiffnesses of 62.508 and 30.736 kPa, respectively (two-tailed Student’s t-test; *p* < 0.001) (Figure 2A). There was not a significant difference between the stiffness of the central and limbal corneal epithelial regions within respective age cohorts (two-tailed Student’s t-test; *p* = 0.753 for 9-week mice and *p* = 0.603 for 27-week mice). The stroma from corneas of 27-week mice was significantly stiffer than those from 9-week mice, with a mean of 244.06 vs. 67.9 kPa respectively (two-tailed Student’s t-test; *p* < 0.001) (Figure 2B). However, there was not a significant difference in basement membrane stiffness between 9- and 27-week mice, with a mean of 6.202 and 5.958 kPa, respectively (two-tailed Student’s t-test; *p* = 0.506) (Figure 2C). These results suggest that the effects of aging on the stiffness of the cornea vary between layers, with the greatest change in the stroma.

### Nine- and 27-week Old Mice Have Significant Differences in Both Short-Term and Long-Term Wound Healing

**FIGURE 3:**
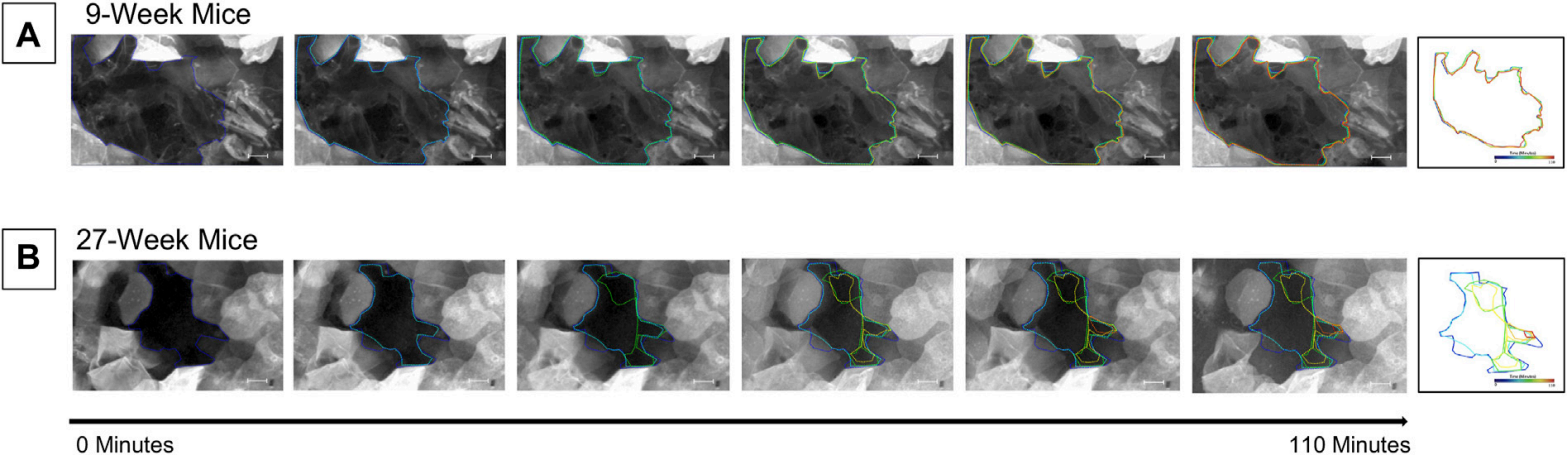
Cells enter the wound bed within 2 h of injury in 27-week but not 9-week old mouse corneas. Enucleated globes were stained with CellMask DeepRed (greyscale) and imaged on the Zeiss LSM 880 confocal microscope to observe wound healing progress for 1 hour and 45 min. *T* = 0 is 15 min after injury. There is a 15-min interval between each image. The border of the wound was traced at each time point using ImageJ and superimposed on the image. Previous wound borders were also superimposed to track changes in the wound border over time. The right-most image depicts the superimposed changes in the wound edge tracked through time. The wound border in the cornea from 9-week mice **(A)** remained approximately the same throughout the duration of the experiment. In the corneas from 27-week mice **(B)** the perimeter of the wound became smaller due to an influx of cells into the wound bed. The data represent three eyes per condition, with each eye from one mouse. Scale bar represents 50 microns.

Short-term wound healing was assessed in corneas from both 9- and 27-week mice. Globes were enucleated, stained with CellMask DeepRed (greyscale) as described, and puncture-wounded using a 25-gauge needle (see methods). Injured globes were mounted into the 3D printed holder and were imaged using the Zeiss LSM 880 confocal microscope using a ×20 objective for 1 h, 45 min at a rate of one frame every 5 min. Imaging began 15 min after injury. Globes were maintained in Keratinocyte Growth Media in an environmental chamber to maintain tissue hydration and viability throughout the duration of the experiment. Analysis of the images using FIJI/ImageJ revealed that the circumference of the corneal wound in the 9-week mice (Figure 3A) remained approximately the same throughout the duration of the experiment. However, the circumference of the corneal wound from 27-week mice was reduced (Figure 3B).

**FIGURE 4:**
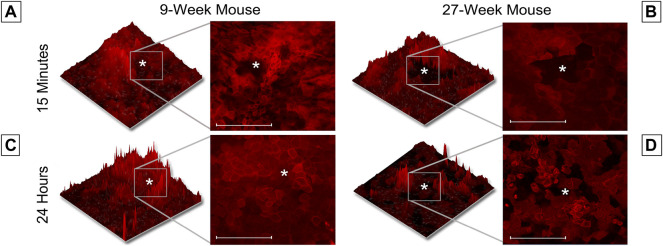
Wound healing occurs more rapidly in 9-week than 27-week mice. Enucleated globes were stained with CellMask DeepRed (red) and imaged using the Zeiss LSM 880 confocal microscope both before and after healing for 24 h in growth media. Images were taken of corneal wounds 15 min or 24 h after injury in both 9-week **(A,C)** and 27-week **(B,D)** mice. In all images, the wound is marked with a white asterisk. Twelve optical sections of one micron each were taken for every condition and Zen software was used to make a 2.5D topographical map of the wounds in the corneas. After 24 h, wounds in 9-week mice had fully closed. Wounds in 27-week mice remained open. Wound depth (Two-tailed Student’s t-test, *p* = 0.999) and diameter (Two-tailed Student’s t-test, *p* = 0.999) were consistent between samples. The data represent three eyes per condition, with each eye from one mouse. Scale bar represents 20 microns.

This unexpected difference in the cellular movement seen in short-term wound healing led us to hypothesize that there would be substantial differences in long-term wound healing between age cohorts. To test this hypothesis, globes were enucleated, injured, and allowed to heal in Keratinocyte growth media at 37° and 5% CO_2_ for 24 h. Representative images of unhealed wounds for 9- and 27-week mice are shown in Figures 4A,B, respectively. In all images, the wound is denoted with a white asterisk. Twelve optical slices were taken for each eye, with an interval of one micron between slices. Using Zen software, slices were compiled and a 2.5D topographical map of the wound after 15 min was generated for corneas from 9-week (Figure 4A) and 27-week (Figure 4B) mice. Corneas from 9-week mice were healed after 24 h (Figure 4C). After 24 h, the corneal wound bed of 27-week mice remained partially exposed (Figure 4D). The topographical map revealed that cells remained at the bottom of the wound bed. Compared to corneas from 9-week mice, evidence for re-stratification was not detected. The results of Figure 4 indicate that the early influx of cells in the 27-week mice (Figure 3B) may not represent a coordinated migration event, as the wound bed remained partially exposed several hours later. Instead, these events together suggest that cells from older mice may move into the wound bed in an unregulated manner. It will therefore not fill the wound bed completely or re-stratify as this is not a wound healing response. A possible etiology of this movement is the above-described changes in epithelial stiffness seen in Figure 2 which could potentially affect cellular motility over a substrate. Another possible cause would be alterations in calcium signaling.

### Calcium Signaling Events Occur in Basal Cells But Not Apical Cells in Young Mice

**FIGURE 5:**
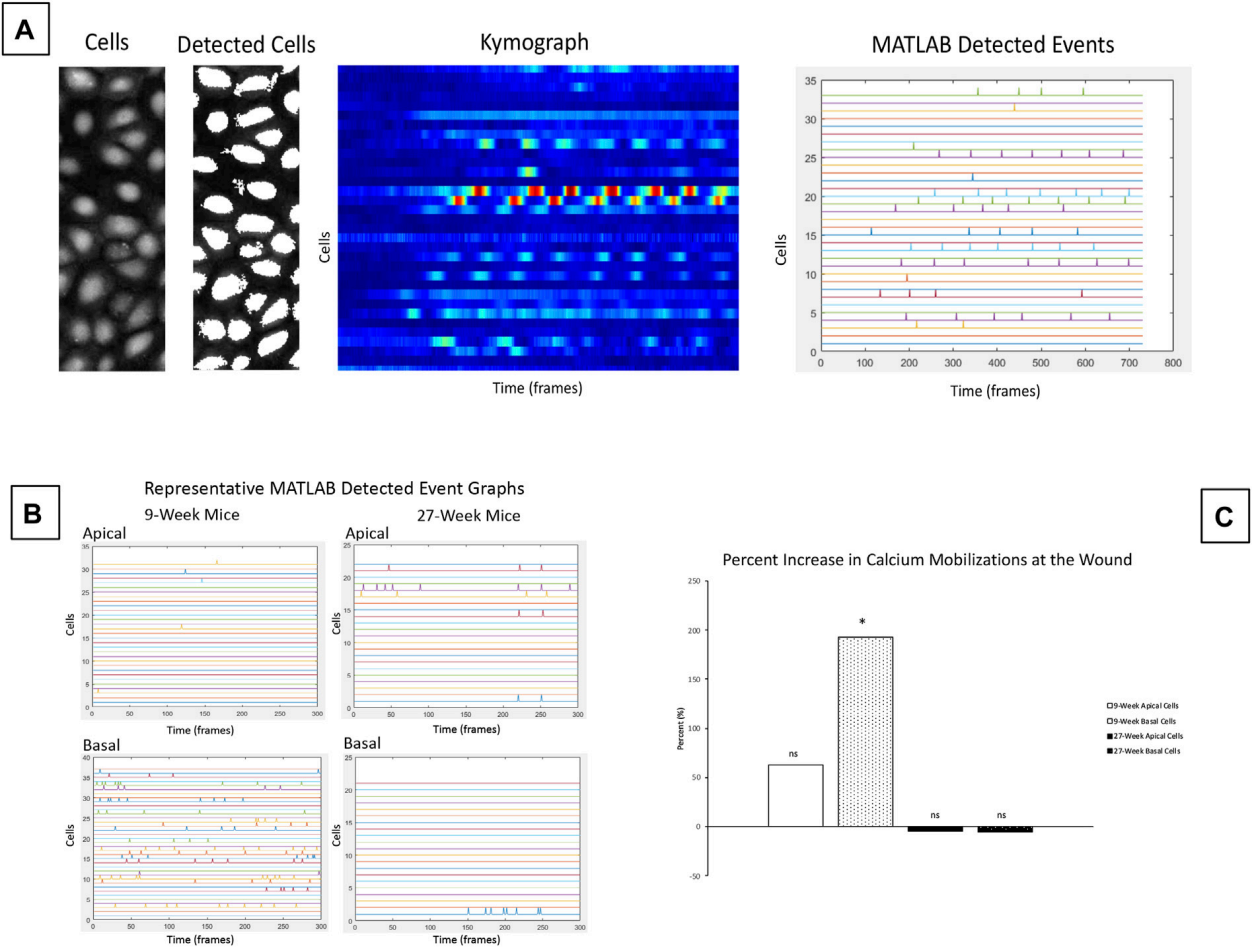
Calcium signaling events occur in basal cells adjacent to the wound in corneas from 9-week mice. Calcium signaling events were monitored in apical and basal layers of the corneal epithelium after injury. **(A)** Schematic of cell-based approach for calcium analysis shows a representative image of cells, and detection of these cells by MATLAB using coordinates from the video. The changes in intensity of each cell over time are shown using a kymograph. MATLAB detected events were identified from the kymograph as changes in intensity greater than a threshold of 40% of maximum intensity. **(B)** Representative MATLAB detected events from basal and apical z-planes of corneal epithelium. **(C)** Mean percent change of the signaling events for apical and basal layers of the age cohorts. The data represent four eyes per condition, with each eye from one mouse. Data are means ± SEM. There were significantly more detected signaling events in the basal cells in 9-week corneas. (Two-tailed paired Student’s t-test comparing signaling events at the wound to background signaling at the same Z-plane; *p < 0.05). No significant differences over background were detected between apical cells from either age cohort or between basal cells from older mice.

The significant differences in cellular motility observed in Figure 3 and Figure 4 led us to hypothesize that there were underlying differences in the calcium signaling profiles of corneas from 9- and 27-week mice. To detect calcium signaling events in basal and apical cell layers, we collected equivalent Z-stacks over time. Enucleated globes were stained with CellMask DeepRed (red) and Fluo-4, AM calcium indicator (green), injured, and immediately imaged. MATLAB scripts were used to identify individual cells within the images, generate kymographs to monitor changes in cell intensity over time, and quantify MATLAB detected signaling events. Representative images of the analysis process are given in Figure 5A. These scripts were applied to videos of signaling events taken from apical or basal cell layers adjacent to a wound in both 9- and 27-week cohorts to quantify the number of signaling events in each condition (Figure 5B). The number of signaling events observed in each layer at the wound edge was compared to background signaling data collected on the same Z. Figure 5C shows the percent increase in calcium mobilizations observed at the wound relative to background levels observed away from the wound for each age cohort and epithelial layer. Basal cell signaling in corneas from 9-week mice was significantly higher than background levels (Paired two-tailed Student’s t-test; *p* < 0.05). There were not significant differences from the background signaling in apical cells from either age cohort, or from basal cells in the 27-week mice.

### Central Corneal Injuries Induce Calcium Mobilizations in the Corneal Limbus

**FIGURE 6:**
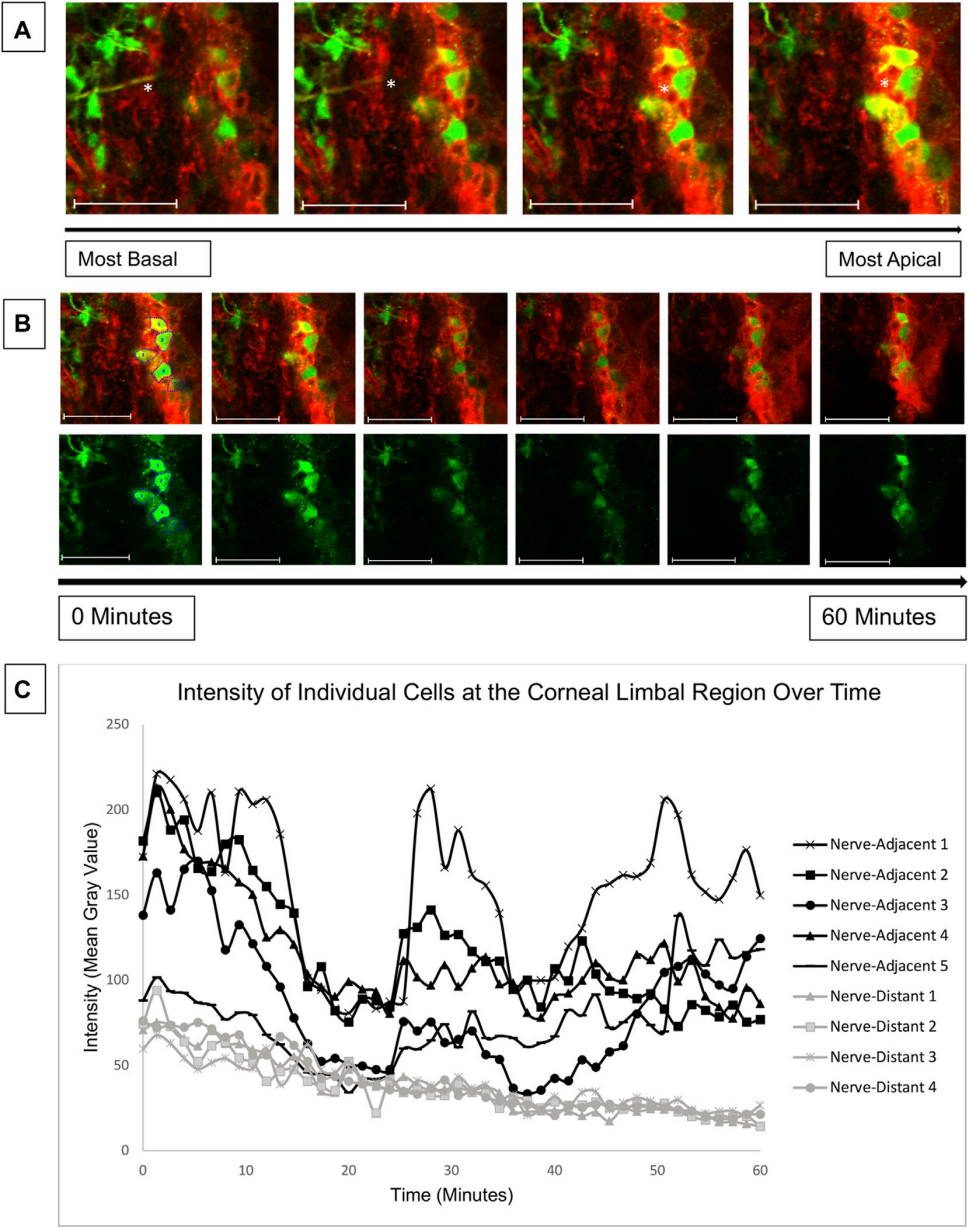
Calcium mobilizations are detected in epithelial cells adjacent to nerves in the corneal limbal region. Enucleated globes from 9 to 13 week mice were stained with CellMask DeepRed (red) membrane stain and Fluo-4, AM (green) calcium indicator, wounded, and imaged in the limbal region of the cornea for 1 h using the Zeiss LSM 880 confocal microscope. Imaging started 15 min after injury (*T* = 0). **(A)** Confocal imaging through the thickness of the corneal limbal region revealed high basal cell activity adjacent to nerves (white asterisk). **(B)** Basal cells were imaged over time at a rate of one frame per 3 s to identify calcium signaling events. The top row depicts tissue stained with CellMask DeepRed and Fluo-4 for cell visualization, and the bottom row depicts the same tissue with Fluo-4 only. **(C)** Quantification of the intensity of the cells labelled in **(B)** over time demonstrates that cells adjacent to the nerve display distinct signaling events at 0, 25, and 42 min after imaging began. At the 25 and 42 min signaling events a previously inactive neighboring cell (Nerve-Adjacent 5) was activated. All cells denoted “Nerve-Adjacent” are within 100 microns of the nerve. The data represent five eyes per condition, with each eye from one mouse. Scale bar is 50 microns.

The results of Figure 5 reveal elevated calcium signaling in the basal layers of young mouse corneas adjacent to a wound. This calcium signaling is initiated by ATP released from injured cells and from the tear film (Klepeis et al., 2001). The ATP acts in a paracrine fashion to bind receptors and generate calcium signaling events. Given the mechanism of action, we hypothesized that an injury at the central cornea could also cause cell signaling at the corneal-limbal interface. Due to the lack of signaling seen at the wound in the central cornea in 27-week mice, we only examined corneas from young mice for our limbal signaling experiments. Globes were positioned in the 3D holder with the limbus facing the cover glass instead of the central cornea as in previous experiments. Imaging began 15 min after injury. Z-stacks revealed that cellular activity in the limbus occurred predominantly in basal cells that were adjacent to nerves (Figure 6A). Representative images taken over 1 hour revealed a number of signaling events in cells overlying the nerve (Figure 6B). Quantification of cellular intensity over time of cells adjacent to (denoted in the first frame of Figure 6B) and distant from the nerve revealed that distinct calcium signaling events occurred in adjacent cells every 20 min (Figure 6C). Recruitment of a previously non-signaling cell, denoted “Nerve-Adjacent 5”, by the active cell “Nerve-Adjacent 3” was observed 25 min after imaging began. This finding suggests that these cells represent a “cluster” of cells, as observed in cell culture models in Lee et al. (2019) but not previously shown in ex vivo tissues. A cluster of cells is defined as a group of neighboring cells that undergo temporally linked calcium signaling events. These signaling events likely mediate cell-cell communication between the cells and are important for coordinated cellular migration. Cells at the corneal-limbal interface that were distant from a nerve did not undergo signaling events nor did they form clusters.

### Pannexin-1 Localization Differs Between Young and Old Mice

**FIGURE 7:**
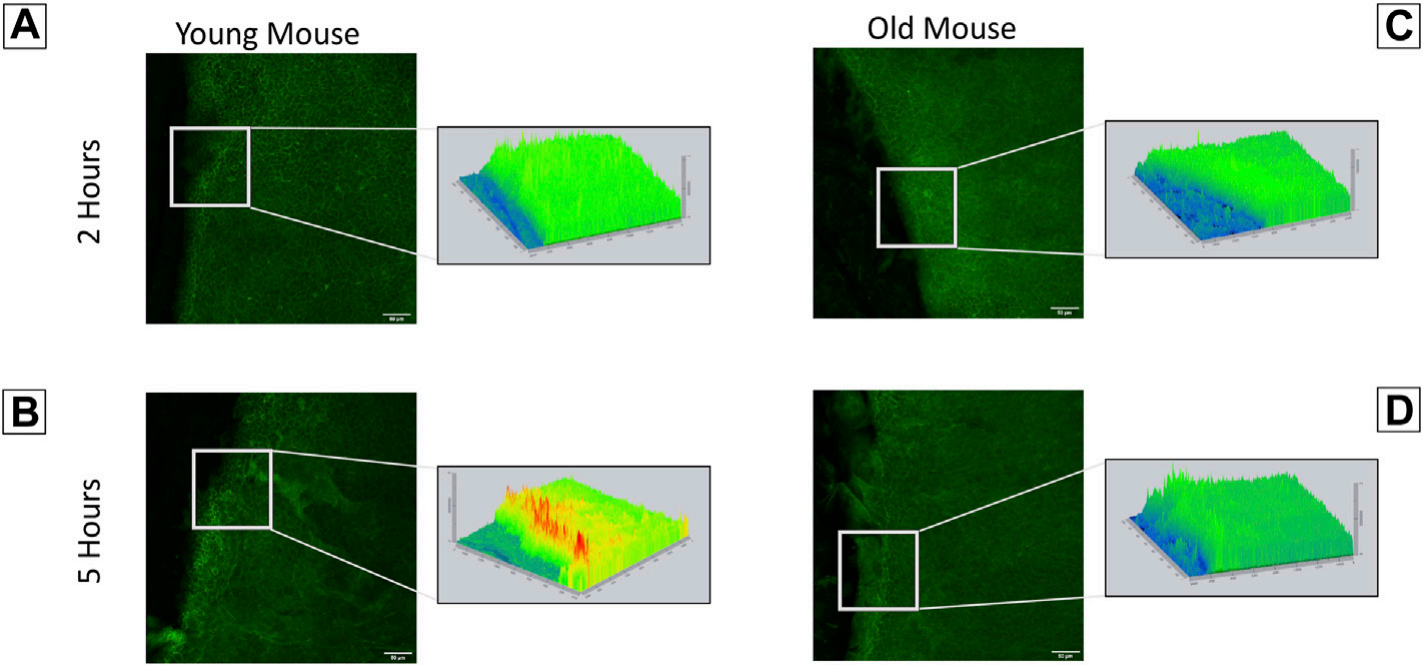
Localization of pannexin-1 is elevated in corneas from younger mice at the wound edge. Enucleated globes were scratch wounded and allowed to heal for two or 5 h. Globes were fixed in 4% paraformaldehyde, corneas were dissected, and immunohistochemistry was performed to visualize localization of pannexin-1 near the wound edge (green). Imaging was performed using the Zeiss LSM 880 confocal microscope. Topographical maps denoting the intensity of staining throughout the region were generated from the imaging data. **(A)** In younger mice, pannexin-1 localization is diffuse at 2 h, with similar expression adjacent to and further away from the wound. This is seen in the topographical map as a relatively flat, green diagram. **(B)** In younger mice at 5 h, the increase in pannexin-1 near the wound edge is detected as red peaks in the topographical map. **(C,D)** Older mice have a similar localization of pannexin-1 throughout the epithelium at both two and 5 h. The data represent three eyes per condition, with each eye from one mouse. Scale bar is 50 microns.

Our findings indicate that corneas from young and old mice have significant differences in their interactions with the substrate, timeline of wound response, and calcium signaling profiles. This led us to speculate that they will have differences in the localization of the ion channel pannexin-1. Immunohistochemistry was performed on corneas from both young and older mice to assess pannexin-1 localization two and 5 hours after injury (Figures 7A–D). Topographical maps colored using a LUT scale were generated to visualize the intensity of staining throughout the region of interest. After 2 h, staining was similar throughout the epithelial sheet in both the young and old mice. At 5 h, there was an increase in pannexin-1 near the edge in young mice, as shown by the intensity image (Figure 7B). A similar increase was not detected in wounded epithelium from older mice. These differences in pannexin-1 localization in older mice together with the decreased calcium signaling events observed in Figure 5 led us to examine the role of pannexin-1 on cellular motility and wound closure in cell culture scratch wound models.

### Inhibition of the Purinergic Receptors P2X7 and P2Y2, and the Ion Channel Pannexin-1 Have Adverse Effects on Wound Healing and Coordinated Cellular Migration in Cultured Human Corneal Limbal Epithelial Cells

**FIGURE 8:**
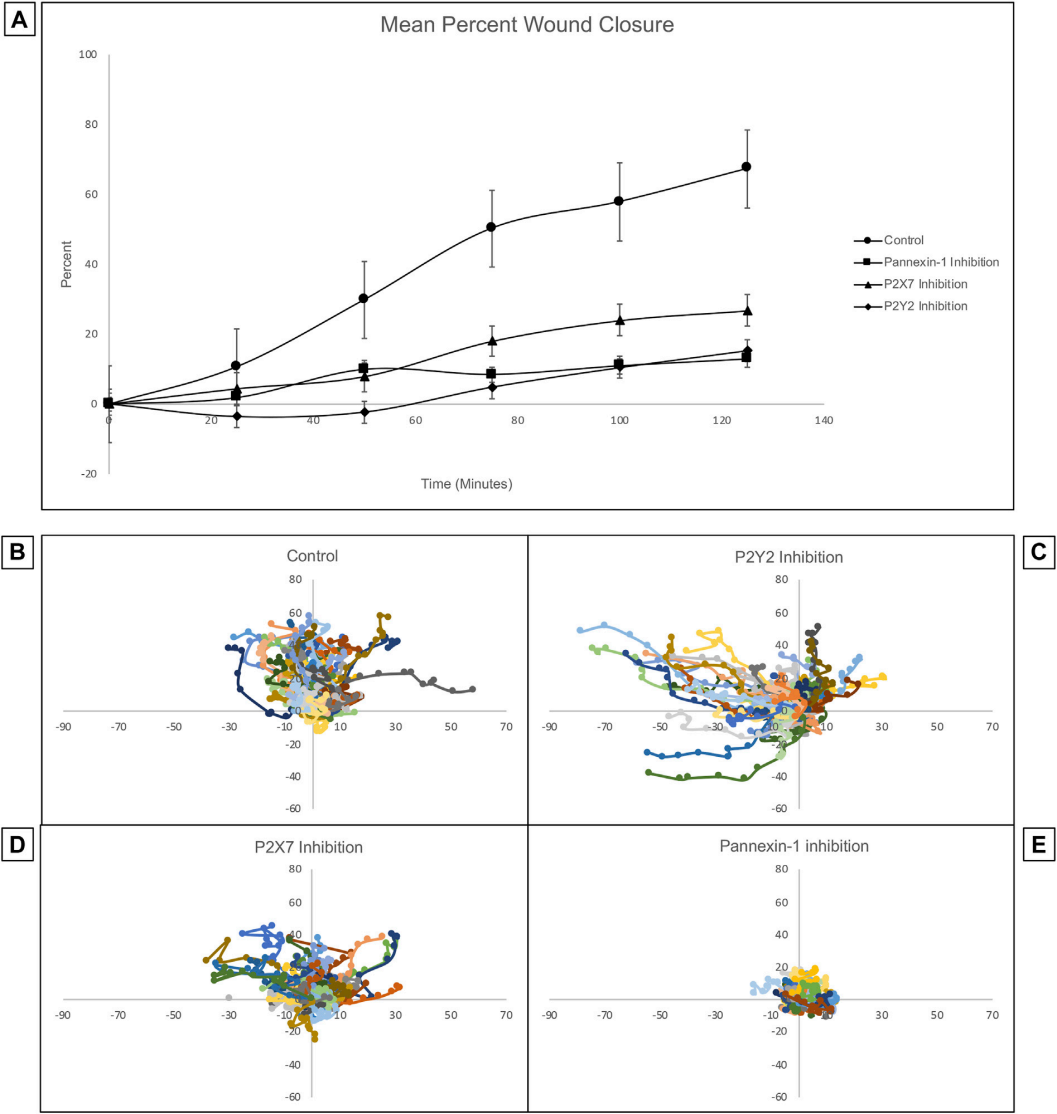
Inhibition of pannexin-1, P2X7, or P2Y2 have different effects on cellular trajectory at the wound edge but all decrease percent wound closure. Confluent Human Corneal Limbal Epithelial cell cultures were pre-incubated in the presence or absence of inhibitors, stained with the SiR Actin, scratch-wounded, and imaged at a rate of one frame per 5 min for 2 h using the Zeiss LSM 880 confocal microscope. **(A)** Inhibition of pannexin-1, P2X7, or P2Y2 led to significantly diminished wound closure at the 2 h time point. Two-tailed Student’s t-tests were performed to compare the percent wound closure of each inhibitor to an uninhibited control (*p < 0.05 for all inhibitors). The path of individual cells at the wound edge was plotted using ImageJ and normalized so that motility towards the wound is represented as movement along the y-axis in the positive direction, and movement in the parallel direction is plotted along the x-axis. Controls **(B)** were scratch-wounded but not pre-incubated with an inhibitor. **(C)** Pre-incubation with ArC 118925XX, a competitive inhibitor to P2Y2, results in uncoordinated and undirected migration. **(D)** Pre-incubation with A438079, a competitive inhibitor to P2X7, causes cells to remain closer to their location of origin than they do in controls. **(E)** Pre-incubation with 10PanX, an inhibitor to pannexin-1, causes cells to remain closer to their position of origin than in both the control and in the P2X7-inhibited conditions. Data represents a minimum of three independent experiments for each condition.

Previous studies of corneas *ex vivo* have demonstrated that the inhibition of pannexin-1 decreased the number of cell-cell signaling events, leading us to hypothesize that the communication in the cornea might be driven by a pannexin-1/P2X7 signaling event (Lee et al., 2019). Confluent Human Corneal Limbal Epithelial cell cultures were pre-incubated in the presence or absence of inhibitors, scratch-wounded, and imaged at a rate of one frame per 5 min for 2 h using the Zeiss LSM 880 confocal microscope. Inhibition of P2Y2, P2X7, or pannexin-1 using the antagonists ArC118925XX, A438079, and 10PanX, respectively led to significantly diminished wound closure (Figure 8A). Mean wound healing after 2 h was 67.18% in the control group, 12.74% for pannexin-1 inhibition, 15.37% for P2Y2 inhibition, and 26.75% for P2X7 inhibition. A two-tailed Student’s t-test was performed to assess differences in percent wound closure between the control and each inhibitor cohort. All inhibitory conditions showed a statistically significant (two-tailed Student’s t-test, *p* < 0.05) reduction in wound closure at the end of the experiment relative to the uninhibited control.

The trajectory of individual cells at the wound edge was plotted using ImageJ and normalized so that movement towards the wound is represented as movement along the y-axis in the positive direction and movement parallel to the wound is represented as movement along the x-axis (Figures 8B–E). The inhibition of P2X7, P2Y2, or pannexin-1 with specific antagonists has different effects on the trajectory of the cells. Cells remain highly motile when P2Y2 is inhibited. However, the movement is undirected and uncoordinated. Instead of moving perpendicular to the wound in a directed migratory event as in controls, the cells tend to move parallel to the wound. This is unproductive for a wound healing response, which requires directed and coordinated movement perpendicular to the wound bed, as observed in the uninhibited control. When P2X7 is inhibited, cell motility is attenuated and maintained directionality. In contrast when pannexin-1 is inhibited, cells remained at the origin compared to controls. Together these results indicate that P2X7 are pannexin-1 are important for coordinating the wound healing response.

## Discussion

As corneal wound healing is predominantly a migratory event, the interactions between the cells and the substrate plays a significant role in the efficacy of wound repair. Nanoindentation experiments of fresh tissue revealed substantial increases in the stiffness of the corneal epithelium and stroma, but not the basement membrane, in 27-week old mice when compared to 9-week old mice. Similar findings have been observed in post-mortem human corneal tissue, which was found to double in stiffness between the ages of 20 and 100 years (Cartwright et al., 2011). In the human cornea, the change in stiffness was found to be due predominantly to changes in collagen fibrils in the stroma (Daxer et al., 1998). Furthermore, changes in human sclera with age have been reported and decreased levels of sulfated glycosaminoglycans were associated with decreased levels of scleral hydration (Brown et al., 1994). Similar modifications may occur in the cornea, and the reduction in sulfation could alter stiffness.

Although changes in stromal stiffness with age are well-characterized, the etiology of the change in stiffness of the corneal epithelium observed in our nanoindentation experiments remains unknown. In the 27-week mouse eye, cells move into the wound bed within 2 h of the injury. However, 24 h after injury the wound bed remains partially exposed and there is no evidence of re-stratification. This is in contrast to the 9-week mouse cornea, which does not experience significant migration into the wound bed within 2 h after injury, but is fully healed after 24 h. The *in vitro* wound closure experiments suggest that purinoreceptors and pannexin-1 mediate different features of cell motility, and it is not known if the altered movement of the cells from the 27-week mice exhibit altered purinoregulation. If rat and murine epithelium have similar expression profiles, this would be unlikely, as one study revealed only minimal changes in P2Y2 expression on the ocular surface of rats over from birth to 1 year of age (Tanioka et al., 2014). Other studies *in vitro* demonstrated that inhibition of P2Y2 abrogated downstream signaling in a number of tissues, but comparisons are difficult as the directionality detected in our *in vitro* assays was not examined (Boucher et al., 2010; Faure et al., 2012; Kehasse et al., 2013; Li et al., 2013). At this time it is not understood why the movement of the corneal epithelium from older mice differs from the younger tissue and it may be a combination of changes in purinoreceptors and matrix proteins.

Furthermore, there was no significant signaling above background levels in either apical or basal cells adjacent to a wound in corneas from 27-week mice. In contrast, corneas from 9-week mice experienced significantly increased signaling relative to background levels in basal but not apical cells adjacent to a wound. While this signaling has been shown to be essential for initiating and coordinating the wound healing process in epithelial cells *in vitro*, this is the first time this has been shown in the cornea (Klepeis et al., 2001; Minns et al., 2016; Lee et al., 2019). Without subsequent calcium signaling events, it is unlikely the cellular movement seen in the 27-week cornea 2 h after injury represents a coordinated cellular migration event. While it is not known why there is decreased signaling in tissue from older mice there are a number of hypotheses that will be tested in future studies. One hypothesis is that there is a change in extracellular ATP released from wounds or during migration, and studies have shown that decreased ATP can alter chemotaxis or directionality of neutrophils (Chen et al., 2006). We will also examine changes in ectonucleotidases, decreased expression of pannexin-1 and other potential candidate proteins, or a decrease in the interaction between P2X7 and pannexin-1. The latter was shown in wounded corneas of an obese mouse (Rhodes et al., 2021).

Although there are few studies investigating motility in intact tissue, this conclusion is supported by extensive studies evaluating cell motility on synthetic substrata. On stiffer substrates, there is less retraction at the edge of the wound prior to movement, and the trailing edge lifts more readily, potentially generating more of a sliding motion (Kuwabara et al., 1976; Pathak and Kumar, 2011; Onochie et al., 2019). We therefore speculate that the cells seen in the wound bed of the 27-week mouse cornea both at 2 and 24 h did not actively migrate, and instead slid into the wound space. (Kuwabara et al., 1976).

There are several potential mechanisms that could explain the altered corneal epithelial stiffness in 27-week old mouse eyes including changes in adhesive proteins, cell-cell junctions, or ion proteins. These changes may underlie the altered calcium signaling and cellular mobility. One hypothesis is that the enhanced epithelial stiffness alters the ability of cells to move as a sheet when they transition from a stationary to a migratory phenotype. Preliminary experiments indicate that ZO-1 and occludin are more diffuse in older mouse corneas. These proteins are essential for maintaining the barrier function of the cornea by linking adjacent apical cells together. ZO-1 is also expressed with paxillin in the basal and wing layers of the rabbit corneal epithelium, where it forms adherens junctions (Sugrue and Zieske, 1997). Cadherin is another component of adherens junctions, and ablation of N-cadherin in mice revealed no adherens junctions and reduced numbers of tight junctions in the corneal epithelium, with severe perturbations in the actin cytoskeleton (Vassilev et al., 2012). In tumors originating from epithelium, a switch from production of predominantly E-cadherin to predominantly N-cadherin has been linked to epithelial-to-mesenchymal transition, and is associated with increased migratory behavior, invasiveness, and likelihood of metastasis (Mrozik et al., 2018). Pathological alterations in tight junction and adherens junction proteins, as well as a significantly increased epithelial stiffness, are also observed in endometriosis (Matsuzaki et al., 2017). Decreased integrity of apical tight junctions and basal adherens junctions with age may lead to the unregulated sliding of cells into the exposed wound bed.

In addition, mechanical forces between the actin cytoskeleton and substrate are critical for successful migration. Pannexin-1 is a mechanosensitive ion channel that binds to F-actin in the carboxy terminus (Bao et al., 2004; Bhalla-Gehi et al., 2010). Inhibition of pannexin-1 in corneas from 12-week mice with 10PanX caused decreases in calcium signaling after injury and alterations at the leading edge with a reduction in lamellipodia (Rhodes et al., 2021). Pannexin-1 localization near the wound edge is elevated in younger mice when compared to older mice 5 h after injury. Additional studies demonstrated that ablation of pannexin-1 in hippocampal neurons of mice caused an increase in F-actin polymerization and expression of actin-associated proteins (Flores-Muñoz et al., 2021). Pannexin-1 is also known to complex with the purinergic receptor P2X7, and inhibition diminishes both calcium signaling events (Lee et al., 2019) and actin bundling (Minns et al., 2016) in cultured human corneal limbal epithelial cells.

One exciting observation was that injuries to the central cornea resulted in signaling of basal cells in the limbal region. It is known that when the corneal epithelium is wounded, ATP released from injured cells and the tear film initiates calcium signaling events (Klepeis et al., 2001). ATP acts in a paracrine fashion and binds to purinergic receptors on cells near the wound (Klepeis et al., 2001; Mankus et al., 2011). Although the concentration of ATP is highest at the wound, and calcium signaling events are most frequent in cells adjacent to the wound, the paracrine mechanism of action could activate more distal cells. Live cell imaging of this region revealed that cells adjacent to nerves displayed calcium mobilizations, while mobilization was not present in cells distant from nerves. This finding suggests that the nerves in the limbal region play a role in generating calcium signaling events in epithelium beyond what would be predicted by paracrine ATP signaling from the wound alone.

A number of studies have demonstrated that the integrity of corneal nerves is essential for effective wound healing. In rat corneas, the density of sensory nerve terminals decreases with age, and at 24 months is approximately 50% as dense as it was at 6 months (Dvorscak and Marfurt, 2008). These findings were consistent between the central and peripheral cornea. More recent studies showed that decreases in intraepithelial nerve density and corneal sensitivity occurred by 24 months of age in mice (Stepp et al., 2018a). Furthermore, enhanced corneal nerve repair can facilitate wound repair (Stepp et al., 2018b). Changes in innervation also occur in corneas of patients with Diabetes Mellitus Type II, who experience neuropathy that worsens with disease progression (Petropoulos et al., 2013). Similar changes are detected in corneas of obese mice, where pannexin-1 was decreased in stromal nerves (Kneer et al., 2018).

In summary, there are age-related changes in the cornea that ultimately result in decreased signaling between basal cells in older mice. While we still don’t understand how the epithelial stiffness alters wound repair, there are a number of experiments that can address this question. The ideal experiments will employ imaging of epithelial-nerve communication at various sites along the cornea and limbal region. Future directions will involve using a hierarchical clustering machine learning algorithm to quantify signaling profiles between corneas from young and old mice, and ultimately in pathological models that exhibit impaired wound healing.

## Data Availability

The raw data supporting the conclusion of this article will be made available by the authors, without undue reservation.
